# Editorial: Transplant Oncology of Liver Malignancies

**DOI:** 10.3389/fsurg.2021.811223

**Published:** 2022-01-05

**Authors:** Cheng-Maw Ho

**Affiliations:** Department of Surgery, National Taiwan University Hospital and College of Medicine, Taipei, Taiwan

**Keywords:** transplant oncology, liver transplantation, hepatocellular carcinoma, malignancy, risk factors

The evolving field of transplant oncology targets at oncological issues related to potential transplant candidates or recipients. For the Research Topic: Transplant oncology of liver malignancies, we narrowed the field down to liver malignancies of which progress had been actively accumulating. Liver transplantation sits in the center of transplant oncology (1) because, compared to other solid organ transplants, it can achieve long-term survival in transplant candidates with malignancies, mainly hepatocellular carcinoma (HCC) (2). However, by definition, transplant oncology can expand to all transplant fields.

Novel risk factors and biomarkers of malignancies that specific and unique to patients in the transplant setting, such as cross-match (3) and graft size (Kim et al.), can be evaluated under this theme. Kim et al. suggested that positive graft weight change during organ solution perfusion indicates poor prognosis in live donor liver transplantation (LDLT) for HCC. These factors may not carry a weight as heavy as the traditional oncological risk factors but could potentially fine-tune the oncological outcomes in transplant setting. Therefore, evidence derived from non-transplant setting is of paramount importance in caring transplant candidates or recipients. For example, preoperative assessment or prediction of microvascular invasion is of significance in selecting a reasonable surgical strategy to prolong patient survival [Zhang et al., (4)].

As the expansion of eligible criteria to liver transplantation for HCC, the outcomes of liver transplantation for primary, recurrent, or down-staged HCCs might be different. Downstaging of HCC to reduce post liver transplant recurrence is another hotspot for investigation [Bhatti et al., (5)]. Bhatti et al. reported their data that LDLT without prior downstaging could reach comparable survival for T2-T4a HCC with lower AFP. Further refining the eligible criteria enable these transplant candidates with HCC (once “outsiders”) to enjoy the benefit of liver transplantation.

Therapeutic role of liver transplantation in liver malignancies other than HCC (cholangiocarcinoma, sarcomas, metastases from neuroendocrine tumors, or colorectal cancer) remains to be precisely defined and requests more real-world data for validation (Finotti et al., Houben et al.).

Because almost all oncological treatments for transplant recipients are off-label use, investigator-initiated trials and real-world experiences are needed to provide solid evidence. Meanwhile, precisely harnessing immunotherapy in the setting of transplant oncology is a vital issue (6).

Looking forward, with the advance of oncology, organ transplantation could become a bridge to further oncological treatment for those who receive limited onco-therapeutic options due to the presence of organ failure. Inversely, development in transplant oncology may enhance precision immunological manipulation of oncological patients without transplant.

I would like to express special thanks to my co-editors: Ye Xin Koh and Nicolas Syn; and reviewers for their excellent work to accomplish this Research Topic collection.

## Author Contributions

The author confirms being the sole contributor of this work and has approved it for publication.

## Conflict of Interest

The author declares that the research was conducted in the absence of any commercial or financial relationships that could be construed as a potential conflict of interest.

## Publisher's Note

All claims expressed in this article are solely those of the authors and do not necessarily represent those of their affiliated organizations, or those of the publisher, the editors and the reviewers. Any product that may be evaluated in this article, or claim that may be made by its manufacturer, is not guaranteed or endorsed by the publisher.
